# Human digital twins in women’s health nursing

**DOI:** 10.4069/whn.2026.05.27.1

**Published:** 2026-06-30

**Authors:** Hyun Kyoung Kim

**Affiliations:** Department of Nursing, College of Nursing and Health, Kongju National University, Gongju, Korea

## Digital twins and human digital twins

Women’s health care increasingly requires personalized, preventive, and data-driven approaches that address women’s diverse biological and psychosocial health needs. In this context, healthcare digital twins (HDTs) have emerged as promising tools for creating virtual representations of patients that can support prediction, monitoring, and precision interventions [1]. HDTs are technologies that collect and analyze individuals’ physiological, pathological, and behavioral data in real time to create virtual representations of humans in digital spaces [1]. Digital twins (DTs) can be traced back to simulation models developed to reproduce the state of the Apollo spacecraft in the 1970s, and the term “digital twin” began to be used in the 2000s [2]. DTs initially developed mainly in industry, where physical systems were mirrored in real time, real-world structures and systems were simulated, predictive models were created, and DTs were applied to industrial processes [2]. More recently, the medical community has implemented virtual hospital spaces to establish systems that efficiently allocate medical resources and staff or to create virtual environments for training medical professionals [1]. DTs can model hospital settings and educational environments because they transmit real-time information about structures, resources, and patients. They can be used for the clinical orientation training of new nurses by accurately reproducing hospital environments such as emergency rooms, trauma centers, and medical records systems [3]. In nursing education and administration, DTs can be used to simulate clinical practice for nursing students. They also enable the real-time monitoring of medical resource flows, helping nursing managers, nursing administrators, and nursing leaders allocate resources and personnel [4]. Recently, in precision medicine, real-time biometric patient data have been integrated and used to predict health outcomes and support decision-making; in precision nursing, patient data have been modeled and used for patient care and for managing personnel and resources [1].

## Features of human digital twins data utilization

The characteristics of HDT data include diverse data sources, data dynamics, big data, real-time data updating, and the use of artificial intelligence (AI) for data analysis. HDTs use diverse data collected extensively from life logging through wearable devices such as smartwatches, smart rings, and smart glasses, as well as ecological momentary assessments, narratives, medical records, and images. Because these data reflect various aspects of human life and health, the amount of data is vast. Creating a digital model from these data makes it possible to update patient information in real time, thereby creating a dynamic and changing mirror world. Big data are stored in the cloud and delivered to end users rather than being stored on personal computers. When data are analyzed and fed back, they form a circular structure in which information is delivered to HDTs through the cloud [5].

## Artificial intelligence machine learning and human digital twins

AI is appropriate for analyzing HDTs because they contain more information than can be analyzed effectively using classical statistics alone [2]. Machine learning analysis using AI is a technology in which computers learn from data and improve performance autonomously. Machine learning aims to build models that predict or classify new data by learning patterns from input data. Research on machine learning began in the 1950s, but recent advances in data availability and graphics processing units have increased computing power and enabled rapid progress [6]. Whereas traditional statistics focus on data analysis and inference, machine learning is a representative method for utilizing AI by building automated predictive models using statistical methods. In other words, machine learning is a representative approach to implementing AI; neural network analysis is a machine learning method that imitates the structure of human neurons; and deep learning refers to neural network analysis using deep, multilayered neural networks [7]. Machine learning includes supervised learning, unsupervised learning, and reinforcement learning [6]. Supervised learning uses input values and labels, unsupervised learning identifies data structures without correct answers, and reinforcement learning identifies optimal behavior through rewards. Machine learning preprocesses data by addressing missing values and identifying outliers, and exploratory data analysis is performed to adjust and normalize feature distributions, such as by using a standard scaler. Machine learning algorithms perform optimized iterative learning that minimizes errors through matrix operations [7]. Typically, data are split into training and test sets for model development and evaluation, and a validation set may also be used for tuning; the exact proportions vary depending on the data and study design [6]. Methods for minimizing the differentiable loss function can be broadly divided into regression algorithms and classification algorithms. Regression algorithms include ridge regression, Lasso regression, and polynomial regression, whereas classification algorithms include decision trees, random forests, logistic regression, k-nearest neighbors, support vector machines, Bayesian methods, and ensemble techniques such as gradient boosting, Extreme Gradient Boosting, and Light Gradient Boosting Machine. Model performance can be evaluated using metrics such as accuracy, precision, recall, specificity, the F1 score, and the area under the receiver operating characteristic curve. If the data are imbalanced, methods such as undersampling, oversampling, and the Synthetic Minority Over-sampling Technique (SMOTE) can be used, along with hyperparameter tuning, to improve model performance [6,8]. Machine learning is used to predict diseases or health problems in HDTs and to determine the effects of interventions [3]. When a neural network model is used, data are entered into the input layer and learned in a way that minimizes the loss function by using nonlinear functions in the hidden layer; the error in the output layer is then calculated, and the prediction result is generated through a backpropagation algorithm [7]. Because deep learning can be designed and trained using multilayered structures with HDT data, it can produce highly precise results and accurately classify or predict health outcomes according to changes in health risk factors [2].

## Human digital twins in precision nursing

HDTs can be used in disease prediction models, allowing virtual patient simulations to be evaluated for effectiveness by applying patient-specific, tailored interventions [9]. In home- and community-based clinical care, HDTs have mainly been developed to manage patients with diabetes and hypertension by reflecting real-time blood glucose and blood pressure signals. A diabetes patient model can model, predict, and diagnose a patient’s condition by replicating information on blood glucose, exercise, diet, sleep, stress, HbA1c, and medication use, and it can determine intervention effectiveness by providing patient-specific interventions. A hypertension patient model can continuously provide modeling, prediction, prognosis, intervention-effect evaluation, and management by transmitting real-time data on systolic blood pressure, diastolic blood pressure, pulse rate, electrocardiography, weight, diet, sleep, and stress [10]. In addition, several studies have attempted to use HDTs to provide customized interventions for patients with cancer [11], evaluate drug effects [12], and manage older adults [13]. An HDT approach was proposed to develop personas of patients with ovarian cancer and their families by integrating clinical data and qualitative insights, thereby better representing patients’ informational, emotional, and care-related needs for education and decision-support purposes [11]. In patients with chronic pain, a personalized HDT model was used to simulate drug responses to transdermal fentanyl therapy, enabling prediction of drug delivery and analgesic effects as well as optimization of dosing strategies [12]. HDTs for personalized management of type 2 diabetes in older adults integrate individual physiological and behavioral data to predict glycemic control and treatment responses, thereby supporting tailored disease management and continuous care for older adults [13].

## Human digital twins and personas

As suggested by analogies such as digital mirror worlds, shadows, twins, virtual selves, and real-time simulations, digital models can create personas, or virtual patients that are updated in real time. Personas can accurately reflect patient data by creating subtypes for each disease group and level of disease severity [14]. It is possible to create a life-course journey map by tracking changes in a patient’s condition over time, allowing the patient’s chronological life course to be observed longitudinally and enabling the tracking of health risk and protective factors. A persona can be assigned a virtual avatar and can communicate with an AI chatbot, which may promote health through medication, exercise, nutritional, emotional, and educational interventions. Using personas, it is possible to develop subtypes that reflect real-world and cultural contexts by subdividing traditional disease categories. Developing diverse personas according to sex, gender, age, ethnicity, race, socioeconomic status, education, environment, and religion can support customized health care for individuals and communities.

## In silico trials and in silico patients

Because clinical trials involving human patients can pose risks to patient safety, ethical verification should be continuously performed to ensure that these trials are not harmful to patients, and considerable effort is required from researchers. However, when HDTs are used as virtual patients, medical records and health data can be replicated, and virtual models can be developed and controlled using these data. Therefore, it is possible to test various combinations of interventions and examine how health outcomes change accordingly. In silico trials are experiments using data and have recently been attempted in the medical field [15]; thus, a paradigm shift in clinical trials is occurring in digital health. In silico trials are expected to bring innovative changes in confirming intervention effects through HDT data analysis in fields such as precision medicine, precision nursing, and precision nutrition.

## Prospects for human digital twins in women’s health

Despite these developments, the use of HDTs in women’s health nursing remains very rare. One previous study that applied HDTs to women’s health nursing developed 10 personas, ranging in age from 45 to 70 years, to provide information and emotional support for patients with ovarian cancer and their families; these personas incorporated specialized information on ovarian cancer, forums, interviews with patients and nurses, and clinical records from three patients [11]. The ovarian cancer personas reflected emotional needs related to complication management, treatment, hormone therapy, communication, high-quality information, sex and intimacy, and end-of-life nursing among patients with stage 3 and 4 ovarian cancer. They were also models that could be used for data analysis through AI [12], suggesting that HDTs could be developed for patients with other female cancers. Because HDTs for diabetes and hypertension have already been developed [10], it would be meaningful to develop HDTs for women with a history of gestational diabetes and gestational hypertension and to analyze these models using AI. It may also be possible to develop HDTs for women with obesity to implement exercise and nutritional interventions as precision nursing strategies and to use pain management HDTs to control labor pain. Because AI technology remains concentrated in male-centered applications [16], women’s HDTs should be developed in ways that account for sex and gender differences. Despite the promise of HDTs, several challenges remain, including the black-box effect of AI analysis, in which decision-making processes are difficult for humans to understand and interpret; poisoning and backdoor attacks, in which malicious training data manipulate AI systems to produce biased or incorrect outcomes; overfitting caused by repeated calculations and excessive training on the same datasets; and data gaps that fail to reflect the full diversity of human experiences [17]. Therefore, HDTs can function as virtual selves or mirror worlds only when women’s health is sufficiently understood and relevant data are adequately and fairly collected. Future HDTs may enable the development of realistic, gender-sensitive, data-driven models that closely reflect women’s actual health experiences and trajectories through machine learning-based analyses of women’s health data. HDTs could support personalized health management across the women’s life span, including birth, menarche, pregnancy, childbirth, the postpartum period, menopause, female cancers, and care for older adults.
